# Lactational Exposure to Polychlorinated Biphenyls, Dichlorodiphenyltrichloroethane, and Dichlorodiphenyldichloroethylene and Infant Neurodevelopment: An Analysis of the Pregnancy, Infection, and Nutrition Babies Study

**DOI:** 10.1289/ehp.0800063

**Published:** 2008-11-10

**Authors:** I-Jen Pan, Julie L. Daniels, Barbara D. Goldman, Amy H. Herring, Anna Maria Siega-Riz, Walter J. Rogan

**Affiliations:** 1 Department of Epidemiology; 2 Department of Maternal and Child Health; 3 Frank Porter Graham Child Development Institute; 4 Department of Biostatistics; 5 Carolina Population Center and; 6 Department of Nutrition, University of North Carolina, Chapel Hill, North Carolina, USA;; 7 Epidemiology Branch, National Institute of Environmental Health Sciences, National Institutes of Health, Department of Health and Human Services, Research Triangle Park, North Carolina, USA

**Keywords:** breast milk, DDE, DDT, lactation, MacArthur-Bates Communicative Development Indices, Mullen Scales of Early Learning, PCBs

## Abstract

**Background:**

Polychlorinated biphenyls (PCBs) and dichlorodiphenyltrichloroethane (DDT) are persistent, bioaccumulative, and toxic pollutants that were broadly used in the United States until the 1970s. Common exposure to PCBs, DDT, and dichlorodiphenyldichloroethylene (DDE), the most stable metabolite of DDT, may influence children’s neurodevelopment, but study results are not consistent.

**Objectives:**

We examined the associations between lactational exposure to PCBs, DDT, and DDE and infant development at 12 months, using data from the Pregnancy, Infection, and Nutrition Babies Study, 2004–2006.

**Methods:**

We measured PCBs, DDT, and DDE in breast milk at the third month postpartum. Lactational exposure of these chemicals was estimated by the product of chemical concentrations and the duration of breast-feeding. Infant development at 12 months of age was measured by the Mullen Scales of Early Learning (*n =* 231) and the Short Form: Level I (infant) of the MacArthur–Bates Communicative Development Indices (*n =* 218).

**Results:**

No consistent associations were observed between lactational exposure to PCBs, DDT, and DDE through the first 12 months and the measures of infant development. However, DDE was associated with scoring below average on the gross motor scale of the Mullen among males only (adjusted odds ratio = 1.9; 95% confidence interval, 1.1–3.3).

**Conclusion:**

Infant neurodevelopment at 12 months of age was not impaired by PCBs, DDT, and DDE at the concentrations measured here, in combination with benefits from long duration of breast-feeding in this population.

Polychlorinated biphenyls (PCBs) are mixtures of synthetic organic compounds that were widely used as insulators, coolants, and lubricants in electrical transformers, capacitors, and hydraulic equipment and as plasticizers in plastic and rubber products. Production of PCBs for industrial and commercial applications in the United States started in 1929 [Agency for Toxic Substances and Disease Registry (ATSDR) 2000]. Dichlorodiphenyltrichloroethane (DDT) was produced in the United States beginning in 1940. DDT was the first organochlorine pesticide in widespread use and was used extensively as an insecticide in agriculture and mosquito control during the 1950s and 1960s [U.S. Environmental Protection Agency (EPA) 2002]. The active ingredient in the commercial DDT sold as an insecticide was *p,p′-*DDT, accounting for 65–80% of the DDT ingredients (ATSDR 2002; Rogan and Chen 2005). Dichlorodiphenyldichloroethylene (DDE) is the most stable metabolite of DDT (Maroni et al. 2000).

PCBs and DDT are both halogenated compounds that were banned in the United States in 1977 and 1972, respectively, because they persist in the environment and bioaccumulate, adversely affecting the ecosystem and possibly human health (U.S. EPA 2002). Although production and use of these two organic pollutants have been banned for decades, they can still be detected in the environment and in the blood and breast milk of the U.S. population [LaKind et al. 2004; National Center for Environmental Health (NCEH) 2005; Needham et al. 2005]. The main exposure route for humans is their diet, most commonly by eating contaminated meat, fish, and shellfish (ATSDR 2000, 2002). For infants, the main exposure route is breast-feeding (ATSDR 2000, 2002).

Infancy is a highly vulnerable period of exposure to these persistent environmental pollutants. Postnatally, synaptogenesis occurs rapidly over the first 2 years (Levitt 2003). Gilmore et al. (2007) found that the neonate’s cortical gray matter of regions for visual and motor function grows rapidly in the first month, and total brain size increases 100% in the first year. Extremely rapid growth in specific areas may increase the vulnerability of the postnatal brain to environmental pollutants during the first year of life. Because brain maturation is not simultaneous in all areas, chemical exposures at different times may cause adverse effects on different developmental domains (Adams et al. 2000; Rice and Barone 2000).

Epidemiologic studies have investigated the associations between exposure to PCBs, DDT, and DDE and infant neurodevelopment for decades, but the findings have been conflicting and remain inconclusive. Most of the previous studies were conducted during the 1980s and 1990s and focused on infant prenatal exposure to PCBs and DDE through placental transfer from the mother. Little is known about the effects of lactational exposure of infants to the low background levels of persistent organic pollutants in this century. Assessing these effects is also complicated by the coexisting beneficial attributes of breast-feeding, both social and nutritional. Long-chain polyunsaturated fatty acids are essential to fetal and neonatal brain development (Clandinin 1999; Clandinin et al. 1980a, 1980b), yet the richest dietary sources of them for mothers, such as fish, may also be sources for the contaminant chemicals. The potential for any harmful effects from persistent organic pollutants in breast milk to be modified by long-chain polyunsaturated fatty acids warrants consideration. Thus, this study was designed to examine associations between the exposure to environmental levels of PCBs, *p,p′-*DDT, and *p,p′*-DDE through breast-feeding and infant neurodevelopment at 12 months of age in central North Carolina in 2004–2006.

## Methods

### Study population

Subjects were participants of the Pregnancy, Infection, and Nutrition (PIN) Babies Study. The study follows children born to women who participated in the PIN3 and PIN Postpartum Studies (Savitz et al. 1999). The PIN3 Study enrolled pregnant women receiving prenatal care at the University of North Carolina Hospitals from 2001 to 2005 before 20 weeks gestation. Mothers completed several self-administered questionnaires and two phone interviews during pregnancy and one brief questionnaire after hospital delivery. In these questionnaires and interviews, they provided details about their health and lifestyle during pregnancy. After delivery, women were invited to continue participation in the PIN Postpartum Study by allowing two in-home interviews at 3 and 12 months postpartum. Seventy-six percent of the eligible women continued postpartum. Details of these studies are available online (PIN 2005).

Beginning in January 2004, participants in the PIN Postpartum Study were invited to enroll their infants in the PIN Babies Study. This study added developmental assessment of the child at 3 and 12 months of age. The 585 eligible children were singletons without major birth defects. Participation in this analysis also required the mother to enroll by 3 months postpartum and to be breast-feeding (*n =* 331). The study protocols of the PIN, PIN Postpartum, and PIN Babies studies have been approved by the Institutional Review Board of the University of North Carolina at Chapel Hill, and written informed consent was obtained from all participants.

### Exposure measurement

Participant women who were still breast-feeding at the time of the 3-month postpartum home visit were asked to provide a breast milk sample. A milk collection kit containing three 1.5-mL tubes, a plastic pipette, and instructions was sent before the scheduled visit. At around 1000 hours on the scheduled visit day, participants were to follow the written instructions to pump both breasts, gently mix the milk extracted, and use the plastic pipette to transfer the milk into three tubes and store the tubes in the freezer until the interviewers arrived. Samples were then transported on ice to –80ºC freezers, where they were stored pending analyses. Breast milk was collected from women who participated between 2004 and 2006.

Breast milk samples were analyzed for *p,p′-*DDT, *p,p′-*DDE, and 35 PCB congeners according to the existing methods at the Organic Analytic Toxicology Branch of the NCEH at the Centers for Disease Control and Prevention (Sjodin et al. 2004). The measurement of selected chemicals in breast milk samples was performed using gas chromatography/isotope dilution high-resolution mass spectrometry using a MAT95 instrument (ThermoFinnigan MAT, Bremen, Germany). The lipid concentration was gravimetrically determined by an analytical balance AX105 Delta Range (Mettler Toledo, Columbus, OH) with an accuracy of ± 10^–4^ g. Each analysis batch contained 16 unknowns, 2 method blanks, and 2 quality control specimens. The between-assay coefficient of variation was normally < 10%. Concentrations reported here were all lipid adjusted. PCB-153 and *p,p′-*DDE were detected in all 304 samples, and *p,p′-*DDT was detected in 292 samples (96%). Total PCBs was calculated as the sum of 18 detectable PCB congeners in > 70% of samples: PCB-66, PCB-74, PCB-99, PCB-105, PCB-118, PCB-138–158, PCB-146, PCB-153, PCB-156, PCB-170, PCB-177, PCB-178, PCB-180, PCB-183, PCB-187, PCB-194, PCB-196–203, and PCB-199. Concentrations lower than the limits of detection (LOD) were imputed to the median of the method LOD of each measurement divided by a square root of 2 (Hornung and Reed 1990).

In addition to the analyses for DDT, DDE, and PCBs, long-chain polyunsaturated fatty acids were measured in the first 175 of 304 total breast milk samples. The fatty acid extraction and assessment were performed by the Collaborative Studies Clinical Laboratory at the University of Minnesota Medical Center, Fairview (Minneapolis, MN). Relative concentrations of docosahexaenoic acid (DHA) and arachidonic acid (AA) were expressed as percentage of total fat.

### Lactational exposure determination

We quantified the duration of breast-feeding to estimate the lactational exposure of the infant to PCBs, *p,p′-*DDT, and *p,p′-*DDE through the first 12 months of life. Infant feeding status was reported during the maternal home interview at 3 and 12 months postpartum. Women were asked to recall their child’s feeding practices for each month, indicating the frequency that they were breast-fed, were fed infant formula, and were fed other types of food. Exclusively breast-feeding was defined as breast-feeding with no other food or liquid; mostly breast-feeding was defined as breast-feeding and feeding of other supplements equal to or less than one time per day; and breast-feeding with supplements was defined as breast-feeding with feeding any other liquids or solids more than one time per day. The number of months representing each type of feeding was summed and used in a lactational exposure metric (LEM).

The LEM was developed to represent the exposure of the infant to each chemical in the first 12 months as follows:


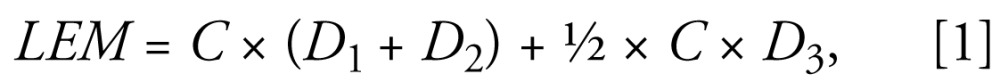


where *C* denotes chemical concentration in breast milk at the third month postpartum (nanograms per gram lipid), *D*_1_ is duration of exclusively breast-feeding (months), *D*_2_ is duration of mostly breast-feeding (months), and *D*_3_ is duration of breast-feeding with other supplements (months). The unit of this estimate is concentration-months [(nanograms per gram)-months]. Using this metric, we assumed breast milk concentration for each chemical to be constant through the lactation period, and breast milk consumption to be decreased by half during the period when breast milk was supplemented with formula, other liquids, or solids.

### Developmental assessment

Infant cognition was measured at 12 months by the Mullen Scales of Early Learning: AGS Edition (Mullen 1995). This standardized assessment instrument was designed to evaluate cognitive and motor functioning of children from birth to 68 months of age in five domains of development: receptive language, expressive language, visual reception, fine motor, and gross motor (Mullen 1995). The Mullen was administered in the home by four trained study staff whose inter-rater reliability was 0.86 [95% confidence interval (CI), 0.70–0.93]. Staff training and clinical oversight were provided by B.D.G., a developmental psychologist. All developmental assessments were conducted without knowledge of exposure status.

Raw scores for each of the five scales are used to derive T scores that take into account the age of the infant, as prescribed by the instrument’s instruction manual (Mullen 1995). Mullen T scores have a mean (± SD) of 50 ± 10. The T scores of receptive language, expressive language, fine motor, and visual reception scales are added together to form the Cognitive T Score Sum, which is used to derive the Early Learning Composite standardized score (mean = 100 ± 15). This composite score provides an overall estimate of the developmental level of the infant. Standardized scores < 1 SD below the mean (i.e., < 40 for T scores on the five individual scales and scores < 85 for the Early Learning Composite) are categorized as below average for the corresponding scale or overall index.

Infant language comprehension and production were also measured at 12 months via parental report, using the Short Form: Level I (for infants) of the MacArthur–Bates Communicative Development Inventories (CDI) (Fenson et al. 2000). The Infant Short Form (for 8- to 18-month-olds) was used to reduce self-administration time and increase the response rate; its scores are highly correlated with the original 396-word full form (*r*= 0.97) (Fenson et al. 2000; Hanson 1994). The instrument prompts parents to endorse whether their child “understands” or both “understands and says” 89 specific words. The raw score for vocabulary comprehension is the sum of the words the child “understands” and “understands and says.” The raw score for infant vocabulary production is the number of words endorsed as “understands and says.”

At the 12-month postpartum home visit, mothers were asked to complete the form and return it in a self-addressed, stamped envelope. Infants’ raw scores for vocabulary comprehension were dichotomized as below the 15th percentile (below average), comparable with the cut points used for the Mullen, versus scores at or above the 15th percentile (average and above average). Production scores were generally low, as would be expected at this age, and did not have sufficient variability to warrant analysis.

### Statistical analysis

All the analyses were conducted using PC-SAS (version 9.1; SAS Institute Inc., Cary, NC). The associations between lactational exposure to PCBs, *p,p′-*DDT, and *p,p′-*DDE and the T scores for each of the Mullen scales were estimated using multivariable linear regression models. The LEMs of PCB-153, PCBs, *p,p′-*DDT, and *p,p′-*DDE were natural log transformed and modeled separately. We used robust regression with least trimmed squares estimation for multiple linear regression models to examine the influence by the outliers of chemical concentrations on beta coefficients (Chen 2002).

We used multivariable logistic regression models to estimate the associations between each chemical and the odds of scoring below average on the Mullen scales and below the 15th percentile scores for vocabulary comprehension of the CDI. The LEMs of PCB-153, PCBs, *p,p′-*DDT, and *p,p′-*DDE were natural log transformed and modeled separately.

To control the positive confounding effects from breast-feeding, the residuals from the models of breast-feeding and Mullen scores were first calculated using simple linear regression and then were included in the multiple linear regression models as dependent variables to estimate the effects of the LEM. Because we were unable to preadjust the confounding effects from breast-feeding for logistic regression models, total months of breast-feeding were included in the models as a continuous variable.

We evaluated other covariates as potential confounders if they had been associated with both lactational exposure to PCBs, *p,p′-*DDT, and *p,p′-*DDE and infant neurodevelopment in the relevant literature but were not an effect of either exposure or outcome. These covariates included maternal age at the start of pregnancy (years), parity (0 vs. ≥ 1), maternal race (nonwhite vs. white), maternal education (≤ 12 years, 13–15 years vs. ≥ 16 years), income as a percentage of the poverty level during pregnancy (100%), and sex of infant (male vs. female). Mother–child pairs missing values for variables in the model were excluded because only 6% were missing any data and their absence did not appear to be related to exposure or outcome status.

The potential for the sex of the infant to modify the measured effects was assessed via interaction terms between sex and the natural log of the LEMs of PCBs, *p,p′-*DDT, and *p,p′-*DDE in the multiple logistic regression models. We assessed the potential for the fatty acids in breast milk to modify the measured effects by dichotomizing the relative concentrations of DHA and AA as < 25th percentile versus ≥ 25th percentile and creating interaction terms between the natural logs of the LEMs of PCBs, *p,p′-*DDT, and *p,p′-*DDE and DHA and AA. The interaction terms were added in the multiple logistic regression models and assessed by the likelihood ratio test. If interaction was indicated (*p* < 0.1), we calculated stratum-specific odds ratios (ORs), 95% CIs, and confidence limit ratios—upper confidence limit divided by lower confidence limit. Because of the smaller sample size of mother–child pairs with known fatty acids concentrations (*n =* 175), the covariates included in the multiple logistic regression models were reduced to major factors, which were total months of breast-feeding, maternal age at the start of pregnancy, and sex of infant.

## Results

Three hundred four (92%) of 331 eligible participants provided samples of breast milk at 3 months postpartum (mean ± SD = 3.5 ± 0.6 months). Two hundred sixty-four of 304 women continued their participation at the 12-month postpartum home visit and provided complete infant feeding information. Among these 264 women, 231 consented to allow their child to be evaluated by the Mullen, and 218 women returned the parent report Short Form of the CDI. The study sample included slightly more male than female infants, and most participant women were white, ≥ 25 years of age, and had > 12 years of education (Table 1). Eighty-eight percent were exclusively or mostly breast-feeding at 3 months postpartum. Most of the women breast-fed for ≥ 6 months and did not smoke in the 12 months postpartum.

In this study population, female infants had higher scores than male infants on the receptive language, expressive language, and fine motor scales of the Mullen and on the Early Learning Composite. Infants who were breast-fed > 9 months compared with ≤ 9 months had higher scores on expressive language, fine motor, and the Early Learning Composite scales. Nonwhite infants and infants whose mothers had ≤ 12 years of education had higher scores on the gross motor scale.

In the multivariable linear regression models, a 2-fold increase in the LEMs of PCB-153, total PCBs, *p,p′-*DDT, and *p,p′-*DDE changed the Mullen Scale T scores and the Early Learning Composite standardized score by < 2 points (Table 2), and all CIs included zero, indicating no difference. Zero to 0.9% outliers were identified in the robust regression models, but none affected the effect estimates.

In the multivariable logistic regression models, a 2-fold increase in the LEM of PCB-153 tended to increase the odds of scoring below average on the receptive language scale, although the association was not statistically significant (Table 3). A 2-fold increase in the LEM of *p,p′-*DDT and *p,p′-*DDE increased the odds of scoring below average on the gross motor scale, the fine motor scale, and the Early Learning Composite score, although all CIs included 1.

Using the norms appropriate for sex and age, 19% of 218 infants scored below the 15th percentile for word comprehension. The infants who were breast-fed > 9 months were less likely to score below the 15th percentile for comprehension. None of other factors showed an association with comprehension. After adjusting for duration of breast-feeding and other covariates in the multivariable logistic regression models, 2-fold increases in the LEMs of PCB-153, total PCBs, *p,p′-*DDT, and *p,p′-*DDE were not associated with CDI scores (Table 4). Using raw scores as continuous outcomes in the multiple linear regression models adjusted for infant sex and age did not change the results (i.e., no association).

Sex of infant did not modify the observed effects in most models (*p*-values of the interaction terms ≥ 0.1). However, male infants who had a 2-fold increase in the LEMs of *p,p′-*DDE were more likely to score below average on the gross motor scale (adjusted OR = 1.9; 95% CI, 1.1–3.3); this association was not observed among female infants (adjusted OR = 0.7; 95% CI, 0.3–1.5) (*p*-value of the likelihood ratio test = 0.02).

The 25th percentile of DHA and AA concentrations were 0.12% and 0.43% of total fat, respectively. The effects of exposure to PCB-153, PCBs, *p,p′-*DDT, and *p,p′-*DDE were generally similar above and below the 25th percentile of each fatty acid, although the effects of DDE on the odds of scoring below average on the Mullen fine motor scale was higher when DHA level was < 25th percentile (OR = 1.9; 95% CI, 0.8–4.8) compared with the level ≥ 25th percentile (OR = 0.7; 95% CI, 0.4–1.4). Because data were sparse, the stratum-specific estimates were unstable; consequently, this study was unable to confirm or interpret potential differential effects.

## Discussion

In this study, lactational exposures to PCBs, *p,p′-*DDT, and *p,p′-*DDE were generally not associated with infant neurodevelopment at 12 months of age, as assessed by the Mullen and the Short Form of the CDI. However, we found that lactational exposure to *p,p′-*DDE through the first 12 months was associated with scoring below average on the gross motor scale among males but not females. The neurologic toxic effects of DDE on male infants in these data were not precise; however, as a known antiandrogen, *p,p′-*DDE can inhibit the biologic effects of androgens, which might differentially affect the development of males. The effect of DDE on the gross motor score modified by sex of infant has not been reported before. Our results suggest that the potential for this interaction should be examined in other large data sets with available data.

Most prior epidemiologic studies used the Bayley Scales of Infant Development (BSID) (Bayley 1993), which included only two scales (mental and psychomotor). We used the Mullen to assess neurodevelopment because the five subscales may help distinguish among finer domains of development. The Mullen Early Learning Composite score has been found to be highly correlated with the Mental Development Index (MDI) of the BSID (*r* = 0.70), and the Mullen gross motor score has also been found to be highly correlated with the Psychomotor Development Index (PDI) of the BSID (*r* = 0.76) (Mullen 1995), which may provide the basis for comparisons of our study results with those of previous studies. However, both the Mullen and the Bayley Scales are limited in their ability to detect subtle physiologic deficits of the brain and nervous system that might be identified with more sensitive clinical assessments.

In a study of children of Mexican farm-workers in California, 1999–2000, prenatal *p,p′*-DDE was found to be associated with infant PDI at 6 months but not at a later age (Eskenazi et al. 2006). A study of a cohort of 92 mother–child pairs in Spain found that an increase in cord blood DDE levels was associated with decrements in MDI and PDI and was also associated with lower scores on the personal–social scale, locomotor scale, and performance scale assessed by the Griffiths Mental Development Scales at 13 months (Ribas-Fito et al. 2003). In a cohort study in Mexico, 2001–2005, an association was found only between maternal DDE serum level in the first trimester of pregnancy and a reduction in infant PDI throughout the first year (Torres-Sanchez et al. 2007). However, neither study investigated postnatal exposure or reported effect measure modification by sex of infant. Three other studies did not observe associations between DDE and infant neurodevelopment (Darvill et al. 2000; Gladen et al. 1988; Rogan and Gladen 1991).

Prenatal exposure to PCBs was associated with lower scores on the PDI at 6–24 months of age in a North Carolina cohort between 1978 and 1982 (Gladen et al. 1988; Rogan and Gladen 1991). A Dutch study (1990–1992) reported delays in motor development at 3 months, but not at older ages, for prenatal exposure (Koopman-Esseboom et al. 1996). In addition, in the Dutch cohort, infants with postnatal exposure to PCBs scored lower on the PDI at 7 months, after controlling for lactational periods (Koopman-Esseboom et al. 1996). In contrast to these two studies, the Collaborative Perinatal Project cohort (in the United States), and cohorts in Germany, Spain, and Japan did not find any associations between infant motor development and either prenatal PCBs (Daniels et al. 2003; Nakajima et al. 2006; Ribas-Fito et al. 2003; Winneke et al. 1998) or postnatal PCBs (Winneke et al. 1998). None of the studies found associations using the BSID MDI (Daniels et al. 2003; Gladen et al. 1988; Koopman-Esseboom et al. 1996; Nakajima et al. 2006; Ribas-Fito et al. 2003; Rogan and Gladen 1991), although the German study found that PCBs in breast milk related to a decrease in infant MDI (Walkowiak et al. 2001; Winneke et al. 1998). The Michigan cohort (Jacobson et al. 1985) and the Oswego cohort (Darvill et al. 2000), both of which used the Fagan Test of Infant Intelligence to assess infant cognition, found a deficit in infant novelty preference associated with prenatal PCBs exposure.

Total PCB concentrations reported here are not directly comparable with those of previous studies because of differences in biospecimens, laboratory methods, and the congeners contributing to the summary estimate of total PCBs concentrations. Longnecker et al. (2003) compared PCB-153 concentrations from different studies standardized to the same base. With a similar approach, the median PCB-153 concentration in our study population would be much lower than in the study with the lowest PCB-153 concentration that was previously reviewed (Longnecker et al. 2003). The *p,p′-*DDE concentration in our study was also much lower than concentrations previously reported in a North Carolina study from 1978 to 1982 [median concentration in milk at 3 months = 2.07 ppm (2,070 ng/g)] (Rogan et al. 1986).

The exposure assignment in this study used the LEM instead of a single chemical concentration at the time of measurement to estimate the total exposure of each infant to PCBs, *p,p′-*DDT, and *p,p′-*DDE through breast-feeding during the first year of life. This metric accounted for both the chemical concentration in breast milk and the duration of breast-feeding. According to the Hooper et al. (2007) study, PCB-153 concentration in breast milk decreased 4% over the first 6 months of breast-feeding; after 6 months the change in PCB-153 concentration was not more than 1% per month. Among 83 women in our study who had concentrations available in both 3-month and 12-month breast milk samples, there was no statistical difference in the percentage change of the concentrations of PCB-153, PCBs, *p,p′-*DDT, and *p,p′-*DDE. These findings support our assumption of a relatively constant concentration throughout the lactation period of interest here. Additionally, under the assumption that infants in the same feeding status group consume similar breast milk volume and that each mother’s milk had similar fat composition, the LEM should be highly correlated with the absolute value of the accumulated dose of the chemicals throughout the first 12 months of life. The LEM is therefore appropriate for estimating the relative effects of chemical exposure. Results using this method may still be affected by the potential for nondifferential exposure misclassification based on our assumptions of constant concentration and recall errors; however, such bias would only minimally shift effect estimates toward the null.

In contrast to the potential detrimental effects of these persistent organic pollutants in breast milk, breast-feeding could convey beneficial effects through enhanced nutrition or through social enrichment. Our study observed that infants who were breast-fed longer scored higher than infants breast-fed for a shorter period on the expressive language and fine motor scales and the Early Learning Composite of the Mullen; this finding of a beneficial effect of breast-feeding on infant cognitive development agrees with that found in previous studies (Anderson et al. 1999). Because most of the women in this study did breast-feed for ≥ 6 months, to further diminish this confounding effect by long-term breast-feeding, we carefully controlled for breast-feeding, and the potential confounding effects from long-term breast-feeding are reduced in the analyses.

Beneficial fatty acids and possibly harmful environmental pollutants unavoidably coexist in breast milk, mainly from maternal dietary sources. It is difficult to know how the beneficial and harmful constituents of breast milk interact to affect development. Although we evaluated the interaction between the pollutants PCBs, DDT, and DDE, and fatty acids DHA and AA in breast milk on infant neurodevelopment, only one interaction term appeared statistically significant at an *a priori* significant level of 0.1 in the multiple logistic regression models. However, the sample size of this study was too small to provide precise effect estimates. Other studies of larger sample sizes are needed to examine these relationships.

Animal studies have shown that position and different degrees of chlorination of PCB congeners may cause different neurochemical effects (Faroon et al. 2001). The *ortho*-substituted PCB congeners, based on the number of chlorine substitutions in the *ortho* positions (2, 2′, 6, and 6′), have been reported to be more active than coplanar PCBs in modifying cognitive processes in animals (ATSDR 2000). However, our study did not find an association between lactational exposure to different *ortho-*chlorinated PCBs groupings and any measurements of infant neurodevelopment (data not shown).

The Mullen scores at 12 months in this study were calculated without the conventional age adjustment for prematurity (infants born < 37 gestational weeks) that is common in clinical practice. In this sample, conventional age adjustment led to artificially elevated T scores for the 18 preterm infants. These scores were significantly higher than those of the 200 full-term infants on the visual reception and fine motor scales and the Early Learning Composite after controlling for sex of infant, parity, maternal race, maternal age, maternal education, poverty level, and duration of breast-feeding. Therefore, we determined that it was better to use actual chronological age in calculating the Mullen scores. The norming sample for the CDI Short Form had excluded babies born at ≤ 34 weeks. We conducted a sensitivity analysis by excluding the three babies in our sample who were born at 34 weeks and one baby at 33 weeks from the models, and the results were not changed. Therefore, we retained those infants in our analyses. This study population was otherwise very similar demographically to the normative sample for the CDI (Fenson et al. 2000).

One of the study limitations is that we were unable to distinguish the effects of lactational exposure to PCBs, *p,p′-*DDT, and *p,p′-*DDE from the effects of prenatal exposure. PCBs and DDE concentrations in breast milk have been shown to be highly correlated with the concentrations in cord plasma (Ayotte et al. 2003; Rogan et al. 1986), so infants exposed to high chemical concentrations postnatally in breast milk were also likely to be exposed to high chemical concentrations prenatally. The other limitation is that most of our study population breast-fed ≥ 6 months, so we had limited sample size to differentiate the effects of short-term, high-concentration exposure to PCBs, DDT, and DDE from the effects of long-term, low-concentration exposure.

Overall, our study did not find lactational exposure to PCBs, DDT, and DDE to be associated with infant neurodevelopment at 12 months. This may be attributed to the combination of low chemical concentrations in breast milk and the beneficial effects of the long duration of breast-feeding in this study population. Larger studies are needed to assess the potential for sex of infant to modify the association between lactational exposure to *p,p′-*DDE and infant gross motor development and to assess the potential effect measure modification on the associations between environmental pollutants in breast milk and infant neurodevelopment by the concentrations of beneficial long-chain polyunsaturated fatty acids in breast milk. This study contributes to the knowledge of potential effects of accumulated exposure to low levels of PCBs, DDT, and DDE through lactation in the first year of life on infant neurodevelopment, taking into account interactions with fatty acids.

## Figures and Tables

**Table 1:** Characteristics of participant mothers who provided breast milk at 3 months in the PIN Babies study and the mother–child pairs included in this study.

Characteristic	Milk samples (*n =* 304)	Mullen subgroup (*n =* 231)	CDI subgroup (*n =* 218)
Maternal characteristics			
Race			
White	262 (86)	204 (88)	194 (89)
Black	18 (6)	12 (5)	9 (4)
Other	24 (8)	15 (7)	15 (7)
Age (years)			
< 25	32 (11)	24 (10)	20 (9)
25–29	88 (29)	63 (27)	61 (28)
30–34	130 (43)	99 (43)	94 (43)
≥ 35	54 (18)	45 (20)	43 (20)
Mean ± SD	31 ± 5	31 ± 5	31 ± 5
Education (years)			
≤ 12	17 (6)	11 (5)	10 (5)
13–15	37 (12)	26 (11)	23 (11)
≥ 16	250 (82)	194 (84)	185 (85)
Mean ± SD	17 ± 2	17 ± 2	17 ± 2
Parity			
0	160 (53)	118 (51)	113 (52)
≥ 1	144 (47)	113 (49)	105 (48)
Percentage of 2001 poverty level during pregnancy			
Mean ± SD	490 ± 199	493 ± 197	513 ± 190
Missing (no.)	5	4	4
Postpartum smoking			
Yes	13 (5)	9 (4)	10 (5)
No	251 (95)	222 (96)	208 (95)
Missing (no.)	40	0	0
Median (range) of chemical concentrations in breast milk (ng/g lipid)^a^			
PCB-153	17 (2–199)	18 (2–199)	17 (2–199)
∑PCBs^b^	77 (9–708)	79 (12–708)	79 (12–708)
*p,p′-*DDT	5 (< LOD–80)	5 (< LOD–80)	5 (< LOD–80)
*p,p′-*DDE	121 (1–2,140)	117 (15–2,140)	117 (15–2,140)
Infant characteristics			
Median (range) of LEM (ng/g lipid-months)^a^			
PCB-153	119 (12–1,194)	118 (12–1,194)	119 (12–1,194)
∑PCBs^b^	546 (64–4,249)	550 (64–4,249)	549 (64–4,249)
*p,p′-*DDT	33 (1–523)	33 (1–523)	33 (1–523)
*p,p′-*DDE	871 (134–19,260)	867 (134–19,260)	864 (134–19,260)
Duration of breast-feeding (months)			
0–< 6	11 (4)	10 (4)	11 (5)
6–9	6 (25)	60 (26)	54 (25)
> 9–12	187 (71)	161 (70)	153 (70)
Missing (no.)	40	0	0
Mean ± SD	10 ± 2	10 ± 2	10 ± 2
Sex			
Male	163 (54)	122 (53)	120 (55)
Female	141 (46)	109 (47)	98 (45)
Preterm birth			
Yes	22 (7)	18 (8)	18 (8)
No	282 (93)	213 (92)	200 (92)
Age at developmental assessment (months)		12 ± 1	13 ± 1
Mullen Scales of Early Learning^a^			
Receptive language		46 ± 8	
Expressive language		54 ± 9	
Visual reception		51 ± 11	
Gross motor		50 ± 12	
Fine motor		51 ± 11	
Composite standard score		101 ± 14	
CDI Words comprehended		29 ± 20	
Comprehension (percentile)			
< 15th			42 (19)
15th–85th			149 (68)
> 85th			27 (12)

Values are no. (%), mean ± SD, or median (range).

a One subject was missing ∑PCBs concentration and the LEM of ∑PCBs, and one subject was missing *p,p′-*DDT concentration and the LEM of *p,p′-*DDT. An additional 40 subjects in the milk sample group were missing the LEM because of missing duration of breast-feeding through 12 months. The following Mullen scores were missing: two for receptive language, two for expressive language, six for visual reception, seven for fine motor, two for gross motor, and eight for the Early Learning Composite.

b ∑PCBs is the sum of the concentrations of PCB-66, PCB-74, PCB-99, PCB-105, PCB-118, PCB-138–158, PCB-146, PCB-153, PCB-156, PCB-170, PCB-177, PCB-178, PCB-180, PCB-183, PCB-187, PCB-194, PCB-196–203, and PCB-199, in which < LOD is treated as median(LOD) divided by a square root of 2.

**Table 2:** Adjusted mean difference (95% CI) in the scores of the Mullen Scales of Early Learning associated with a 2-fold increase in the LEMs.^a^

	Receptive language	Expressive language	Visual reception	Gross motor	Fine motor	Early Learning Composite
PCB-153	0.0 (–1.2 to 1.2)	0.7 (–0.6 to 2.1)	0.5 (–1.2 to 2.2)	0.0 (–1.9 to 1.8)	1.1 (–0.5 to 3.0)	1.4 (–0.6 to 3.4)
∑PCBs	0.1 (–1.2 to 1.3)	0.8 (–0.6 to 2.2)	0.3 (–1.6 to 2.1)	–0.3 (–2.3 to 1.7)	1.2 (–0.5 to 3.0)	1.4 (–0.7 to 3.5)
*p,p′-*DDT	0.1 (–0.8 to 1.0)	–0.1 (–1.1 to 1.0)	–0.1 (–1.4 to 1.2)	–1.3 (–2.7 to 0.1)	0.3 (–0.9 to 1.5)	–0.2 (–1.7 to 1.3)
*p,p′-*DDE	–0.2 (–1.3 to 1.0)	–0.5 (–1.8 to 0.8)	0.3 (–1.4 to 2.0 )	–1.6 (–3.4 to 0.2)	–0.2 (–1.8 to 1.4)	–0.3 (–2.2 to 1.7)

MD, mean difference.

a Adjusted for maternal age at the start of pregnancy (years), parity (0 vs. ≥ 1), maternal race (nonwhite vs. white), maternal education (≤ 12 years, 13–15 years vs. ≥ 16 years), poverty level during pregnancy, and sex of infant (male vs. female).

**Table 3:** Adjusted OR (95% CI) of being scored below average in the Mullen Scales of Early Learning for a 2-fold increase in the LEMs.^a^

	Receptive language	Expressive language	Visual reception	Gross motor	Fine motor	Early Learning Composite
PCB-153	1.3 (0.8–2.0)	0.8 (0.4–1.6)	0.9 (0.6–1.3)	1.0 (0.7–1.5)	0.9 (0.6–1.3)	0.8 (0.5–1.4)
∑PCBs	1.3 (0.8–2.0)	0.9 (0.4–1.8)	0.9 (0.6–1.4)	1.1 (0.7–1.6)	0.8 (0.5–1.3)	0.8 (0.5–1.5)
*p,p′*-DDT	0.8 (0.6–1.1)	1.0 (0.6–1.7)	1.0 (0.7–1.3)	1.4 (0.9–2.0)	1.4 (1.0–2.0)	1.4 (0.9–2.2)
*p,p′*-DDE	1.1 (0.7–1.6)	1.3 (0.7–2.5)	0.9 (0.6–1.4)	1.4 (0.9–2.2)	1.3 (0.9–2.1)	1.4 (0.8–2.2)

a Adjusted for duration of breast-feeding (months), maternal age at the start of pregnancy (years), parity (0 vs. ≥ 1), maternal race (nonwhite vs. white), maternal education (≤ 12 years, 13–15 years vs. ≥ 16 years), poverty level during pregnancy, and sex of infant (male vs. female).

**Table 4:** Adjusted OR (95% CI) for the infant short form of the MacArthur CDI associated with a 2-fold increase in the LEMs.^a^

	Comprehension < 15th percentile
PCB-153	1.0 (0.7–1.6)
∑PCBs	1.0 (0.6–1.6)
*p,p′-*DDT	1.0 (0.8–1.4)
*p,p′-*DDE	1.0 (0.7–1.5)

a Adjusted for duration of breast-feeding (months), maternal age at the start of pregnancy (years), parity (0 vs. ≥ 1), maternal race (nonwhite vs. white), maternal education (≤ 12 years, 13–15 years vs. ≥ 16 years), poverty level during pregnancy, and sex of infant (male vs. female).
